# Toward the sustainability of health interventions implemented in sub-Saharan Africa: a systematic review and conceptual framework

**DOI:** 10.1186/s13012-016-0392-8

**Published:** 2016-03-23

**Authors:** Juliet Iwelunmor, Sarah Blackstone, Dorice Veira, Ucheoma Nwaozuru, Collins Airhihenbuwa, Davison Munodawafa, Ezekiel Kalipeni, Antar Jutal, Donna Shelley, Gbenga Ogedegebe

**Affiliations:** 1 Department of Kinesiology and Community Health, College of Applied Health Sciences, University of Illinois Urbana-Champaign, Champaign, IL USA; 2 School of Medicine, New York University, New York, NY USA; 3 Saint Louis University, Saint Louis, MO USA; 4 World Health Organization Regional Office for Africa, Brazzaville, Congo; 5 West Virginia University, Morgantown, WV USA; 6 Department of Geography, University of Illinois Urbana-Champaign, Champaign, Il USA

**Keywords:** Sustainability, Implementations, Health interventions, Sub-Saharan Africa

## Abstract

**Background:**

Sub-Saharan Africa (SSA) is facing a double burden of disease with a rising prevalence of non-communicable diseases (NCDs) while the burden of communicable diseases (CDs) remains high. Despite these challenges, there remains a significant need to understand how or under what conditions health interventions implemented in sub-Saharan Africa are sustained. The purpose of this study was to conduct a systematic review of empirical literature to explore how health interventions implemented in SSA are sustained.

**Methods:**

We searched MEDLINE, Biological Abstracts, CINAHL, Embase, PsycInfo, SCIELO, Web of Science, and Google Scholar for available research investigating the sustainability of health interventions implemented in sub-Saharan Africa. We also used narrative synthesis to examine factors whether positive or negative that may influence the sustainability of health interventions in the region.

**Results:**

The search identified 1819 citations, and following removal of duplicates and our inclusion/exclusion criteria, only 41 papers were eligible for inclusion in the review. Twenty-six countries were represented in this review, with Kenya and Nigeria having the most representation of available studies examining sustainability. Study dates ranged from 1996 to 2015. Of note, majority of these studies (30 %) were published in 2014. The most common framework utilized was the sustainability framework, which was discussed in four of the studies. Nineteen out of 41 studies (46 %) reported sustainability outcomes focused on communicable diseases, with HIV and AIDS represented in majority of the studies, followed by malaria. Only 21 out of 41 studies had clear definitions of sustainability. Community ownership and mobilization were recognized by many of the reviewed studies as crucial facilitators for intervention sustainability, both early on and after intervention implementation, while social and ecological conditions as well as societal upheavals were barriers that influenced the sustainment of interventions in sub-Saharan Africa.

**Conclusion:**

The sustainability of health interventions implemented in sub-Saharan Africa is inevitable given the double burden of diseases, health care worker shortage, weak health systems, and limited resources. We propose a conceptual framework that draws attention to sustainability as a core component of the overall life cycle of interventions implemented in the region.

## Introduction

Sub-Saharan Africa (SSA) is facing a double burden of disease with a rising prevalence of non-communicable diseases (NCDs) while the burden of communicable diseases (CDs) remains high. For example, by the end of 2013, an estimated 24.7 million people in SSA were living with HIV/AIDS, with women accounting for 58 % of all people living with HIV in the region [1]. Sub-Saharan Africa continues to bear the heaviest burden of malaria, with 80 % of 219 million cases and 90 % of deaths occurring in the region [2]. In addition, of the 8.6 million new incident cases of tuberculosis, a disease that is both curable and preventable, SSA had approximately 27 % of the cases [3]. Similarly, due to rapid epidemiological transitions characterized by increasing urbanization and changing lifestyle factors [4], the prevalence of NCDs such as cardiovascular diseases and diabetes is also on the rise in SSA [4–6]. According to the World Health Organization’s global health estimates, NCDs are the second leading cause of death in SSA [7–9]. In 2011, NCDs accounted for 30 % of the 9.5 million deaths, and 25.8 % of the 675.4 million disability-adjusted life years (DALYs) recorded in Africa [9, 10]. Available evidence suggests that the number of people in SSA with hypertension, a major risk factor for cardiovascular diseases, will increase by 68 % from 75 million in 2008 to 126 million in 2025 [11]. Furthermore, about 27.5 million people currently live with diabetes in Africa [11]. It is estimated that by 2030, 49.7 million people living with diabetes will reside in Africa [11]. Obesity, a well-known risk factor for many chronic diseases, is also on the rise in SSA with 20–50 % of urban populations in Africa currently classified as overweight or obese [12].

The growing double burden of diseases in the region has led to the reexamining of a long-standing debate in international development for health: sustainability of donor-funded interventions [13]. For many decades, funders and implementers of health interventions have asked the question “what happens among individuals, families, communities or health care systems when donor funding for implementations expires” [14, 15]. This question is especially pertinent for SSA where despite incredible gains in health achieved over the past 20 years (i.e., sharp declines in death among children under five), as noted previously, the continent continues to face a disproportionate share of the global disease burden. In light of the decline in donor funding, funders and policymakers have also become concerned with how to most effectively allocate limited resources, recognizing that intervention implementations which require substantial resources, are meaningless without successful long-term use [16, 17]. As a result, sustainability has become an important global target to achieve [18]. Yet in SSA, despite years of substantial assistance, with the proliferation of numerous actors involved in assisting countries to achieve global disease control targets, the conceptualization of sustainability has received remarkably little critical attention [13]. This paper moves beyond the perspective of intervention implementation in the region to explore sustainability so as to understand how or under what conditions [19] effective interventions implemented in SSA are sustained.

Intervention sustainability is defined in the current literature by various scholars including Scheirer and Dearing [14] who defined sustainability as the “continued use of intervention components and activities for the continued achievement of desirable health outcomes within the population of interest.” An intervention is considered sustainable when its relevant activities and resources continue in the direction of its primary objectives [20]. Chambers and colleagues [16] suggested that sustainability relates to the extent that these interventions can continue to be delivered over time and institutionalized within settings, with necessary capacity built to support their delivery. Shediac-Rizkallah and Bone [21] who offered one of the best definitions described sustainability as falling into one of the three components: (1) continued benefits to those who received health services when the program started and to new participants when the supporting funds are discontinued; (2) continued implementation of a program activities in an organization following the discontinuation of the program financial support; and (3) community empowerment to improve their health by continuing the activities of a finished program. Together, these measures, they argued, allow one to plan for “*what* is to be sustained, *how* or *by whom, how much* and *by when”* [21].

In 2012, Stirman and colleagues examined, through a systematic review of empirical literature, the sustainability of new and innovative programs. Despite the importance of sustainability and its relevance for SSA, the review reported only seven studies conducted in sub-Saharan Africa [22]. Since that review, a significant amount of research on the sustainability of health interventions has been undertaken in the region. However, there is an increasing debate and questions about persistent limitations of potential contributions that researchers could and should be making with sustaining interventions implemented in SSA [23, 24], given decades of significant assistance to the region [15].

Notwithstanding, there are various reasons why increased attention is warranted for sustainability of health interventions, particularly in SSA. For instance, SSA bears a major share of the global burden of diseases with the least resources both financial and human capital to address these challenges [4]. Shediac-Rizkallah and colleagues [21] suggested that termination of an intervention, particularly due to expiration of funding, is counterproductive when the disease or health outcome remains or recurs. Many projects incur significant unexpected start-up costs in human, fiscal, and technical resources, only to see funds expire prior to achievement of predicted potential [21]. Funders of these interventions also want to know whether their investments lead to longer-term beneficial outcomes or fade away after the funding is spent [14, 20]. While sustainability is a desired outcome of effective implementation, there has been little research-based evidence in this area, nor is there any “how to do it” empirical systematic review on sustainability of health interventions in sub-Saharan Africa. The purpose of this study was to conduct a systematic review of empirical literature to explore how health interventions implemented in SSA are sustained. Additionally, we sought to explore how sustainability was defined, the types of methods used, timeframe assessed, and outcomes measured and reported as well as factors identified as potential facilitators or barriers to the sustainability of interventions implemented in the region. Our paper is another addition to the growing body of literature that attempts to inform an agenda for research, funding, and polices on sustainability of health interventions, particularly those implemented in low-resource settings like SSA.

## Methods

Our systematic review addressed the following questions:

1. How is sustainability defined in health interventions implemented in sub-Saharan Africa?
2. Are there any factors (including positive or negative factors) that influence the sustainability of health interventions in the region.

Based on a previous systematic review conducted by Stirman and colleagues [22], we employed a narrative synthesis to extend the current review to specifically consider the sustainability of health interventions implemented in sub-Saharan Africa. We initially set out to systematically examine only studies describing initiatives to promote the systematic uptake of evidence-based interventions into practice and policy to improve health. However, discovering that the review by Stirman and colleagues yielded only seven studies [22], we expanded our scope to include any literature on sustainability of health interventions and programs conducted in sub-Saharan Africa.

### Search strategy

We searched MEDLINE, Biological Abstracts, CINAHL, Embase, PsycInfo, SCIELO, Web of Science, and Google Scholar using the following search terms: (Sub-Saharan Africa OR Central Africa OR Cameron OR Central African Republic OR Central Africa OR Chad OR Congo OR Democratic Republic of the Congo OR Equatorial Guinea OR Gabon OR Eastern Africa OR Burundi OR Djibouti OR Eritrea OR Ethiopia OR Kenya OR Rwanda OR Somalia OR Sudan OR Tanzania OR Uganda OR Southern Africa OR Angola OR Botswana OR Lesotho OR Malawi OR Mozambique OR Namibia OR South Africa OR Swaziland OR Zambia OR Zimbabwe OR Western Africa OR Benin OR Burkina Faso OR Cape Verde OR Cote d’Ivoire OR Ivory Coast OR Gambia OR Ghana OR Guinea OR Guinea-Bissau OR Liberia OR Mauritania OR Niger OR Nigeria OR Senegal OR Sierra Leone OR Togo) AND (sustainable OR sustainability OR “capacity building”) AND (Health interventions OR intervention studies OR evidence-based practice OR evidence-based medicine). We focused mainly on the term “Sustainability*”* because it a global term that appears to better capture the dynamic process involved in program continuation, incorporating notions such as permanence and time and the broad range of its potential form than the notion of similar concepts such as “institutionalization” or “routinization” [21]. We also modified our search strategy using previously conducted systematic reviews on sustainability as a guide [22], by extending their search to incorporate only studies conducted in SSA so as to provide evidence on the current state of the research literature on sustainability of interventions implemented in the region. In addition, reference lists of included studies and available reviews were checked for further possible studies.

### Analytical framework: defining sustainability

**Fig. 1:**
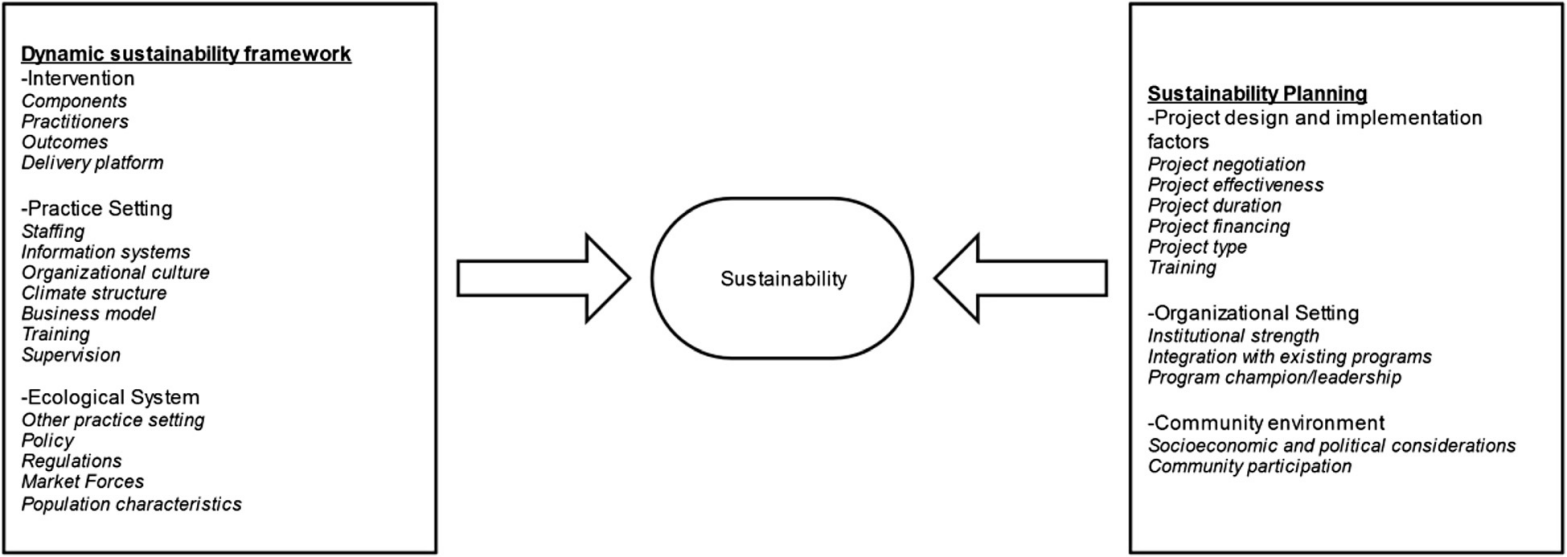
Conceptual framework: defining sustainability

Figure 1 illustrates our definitions of sustainability which is guided by Chambers and colleagues [16] Dynamic Sustainability Framework and Shediac-Rizkallah and Bone’s [21] framework or guidelines for sustainability planning. Dynamic Sustainability Framework was chosen due to its emphasis on the following major elements for sustainability: the intervention, the context in which the intervention is delivered, and the broader ecological system within which health and health care systems exist and operate. Distinct from other models of sustainability, this framework considers these elements over time. It is also highlights the need for continuous assessment of the intervention so as to allow practitioners to make informed decisions about how best to utilize existing interventions and allow for potential enhancements to the interventions to be made and shared, while offering better information on which to make decisions to cease delivering interventions that do not have benefit. We also used Shediac-Rizkallah and Bone’s [21] framework for conceptualizing sustainability as it includes: (1) the characteristics of the project design and implementations; (2) factors within the organizational setting; and (3) factors in the broader community level. These definitions are presented here as a starting point for summarizing similar findings across studies conducted in SSA.

### Inclusion and exclusion criteria

Our inclusion search criteria were as follows: (i) any peer-reviewed studies that addressed sustainability of health interventions implemented in sub-Saharan Africa up until May 2015, there was no limit on the start date of the publication search given the paucity of data from SSA as identified in previous systematic reviews [22]; (ii) provided definitions of sustainability using existing definitions of sustainability, such as those provided by the Dynamic Sustainability Framework [16] or Shediac-Rizkallah and Bone’s [21]; (iii) studies with information on the status of the intervention during or after the initial implementation efforts or funding has ended; and (iv) the continuation of the intervention, whether or not their primary focus was sustainability, with accounts on adaptation or lessons learned.

Exclusion criteria (as adapted from Stirman et al. [22]) were as follows: (i) publications that did not examine sustainability using any quantitative or qualitative research methodologies; (ii) studies with no information on follow-up of individuals after initial implementation efforts; (iii) studies with insufficient information to determine whether inclusion or exclusion criteria were met (e.g., ambiguity or failure to report the timeframe during which measures were collected as well as limited information on project design and implementation characteristics, or aspects of the organizational or the broader community contexts that disproportionately deter or encourage sustainability). Contrarily to Stirman and colleagues [22], and given both financial and human resource constraints often observed in sub-Saharan Africa [25, 26], we considered whether initial sustainability efforts were ongoing during the time period of the intervention prior to expiration of funds.

### Data extraction and appraisal

The titles and abstracts were screened, and the full papers of potentially relevant studies were obtained. Two authors independently assessed the full papers for eligibility and extracted data on study design, sample characteristics, and their findings. Methodological quality was assessed using the critical appraisal checklist for public health [27] which determines the quality of the studies by assessing the validity, completeness and transferability of the data as they relate to the study question, key aspects of the methodology, possible public health implications of the key results, and the implications for implementation research..

### Analysis

Narrative synthesis was used to analyze each retrieved paper. It refers to “an approach to the systematic review and synthesis of findings from multiple sources and relies primarily on the use of words and text to summarize and explain the findings of the synthesis” [27]. Narrative synthesis is used when statistical meta-analysis or another specialist form of synthesis (such as meta-ethnography for qualitative studies) is not feasible particularly due to extreme heterogeneity in the methodological descriptions of available studies [27]. A narrative synthesis was appropriate for this systematic review given an initial scoping that revealed that the literature was too heterogeneous to permit a meta-analysis [4]. It included the following four steps: (1) developing (and/or) identifying a theoretical model, (2) developing a preliminary synthesis, (3) exploration of relationships in the data, and (4) assessment of the robustness of the synthesis.

For step 1, we used the PEN-3 cultural model which is a model designed originally for the study of health behaviors and interventions in the sub-Saharan Africa context [28]. The model has been used previously to examine the impact of context such as culture on health outcomes [28]. We used the model here as a guide to examine positive, unique, and/or negative factors that may influence the sustainability of health interventions in sub-Saharan Africa. For step 2, we used content and thematic analysis to conduct the preliminary synthesis of the retrieved literature. Specifically for each paper that met the inclusion criteria, we extracted the following data: country, type of intervention or program conducted, definition of sustainability, length of time for assessment of sustainability, and the analytical approach used to measure sustainability, as well as results of findings. Two authors independently conducted this tabulation and compared coding decisions to maximize reliability. In the case of disagreement, a third author was brought in and the three authors discussed the coding until a consensus was reached and approved by all parties. The key terms and components of sustainability were then extracted for thematic analysis, to identify themes occurring within the data. Using relevant extracts from text retrieved from available papers, we grouped the themes into categories that highlighted facilitators (positive), unique (existential), and/or barriers (negative) influencing sustainability of health interventions implemented in sub-Saharan Africa. For step 3, efforts were made to examine differences both within and between the data of included studies. We also constructed a table to show where there was overlap between studies. Finally for step 4, we assessed the robustness of our synthesis to provide an assessment of the strength of the evidence retrieved, the conclusions drawn, and the generalizations of findings. We accomplished this through the use of the critical appraisal checklist for public health as a guide and by placing the findings in the context of wider literature, such as Stirman and colleagues [22] review on the sustainability of health interventions and programs.

## Results

**Fig. 2:**
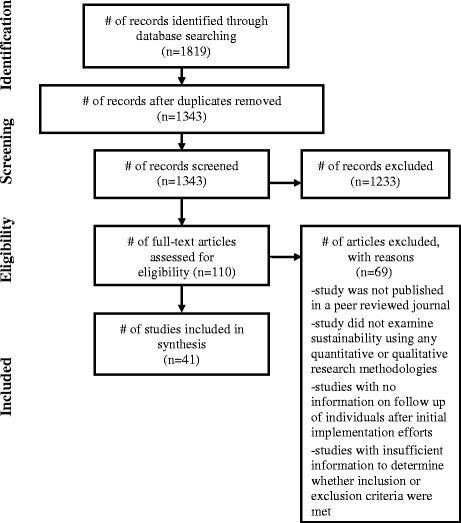
PRISMA diagram of article selection

The flow chart of the search results are presented in the Preferred Reporting Items for Systematic Reviews and Meta-Analyses (PRISMA) diagram in Fig. 2. The search identified 1819 citations, and following removal of duplicates and our inclusion/exclusion criteria, only 41 papers were eligible for inclusion in the review.

### Area of study

As shown in Table 1, which includes a summary of the 41 studies included in this review, the five regions of SSA were fairly represented, with nine (22 %) from Southern Africa countries, seven (17 %) from West Africa, seven (17 %) from East Africa countries, three (7 %) from Central African countries, and one from North Africa. Six papers had more than one country represented. In total, 26 countries were represented in this review, with Kenya and Nigeria having the most representation of available studies examining sustainability. Of note, majority of these studies (30 %) were published in 2014, with the earliest record in 1996.

**Table 1:** Summary of available studies examining sustainability of health interventions implemented in sub-Saharan Africa

Reference/context	Theory or framework	Intervention	Definition of sustainability in frameworks used	Timeline of project (assessment year)	Analytical approach	Results
1. Abbey et al. (2014) Ghana	Ecological behavior model	Community-based fever management	Retention of volunteer CHWs	2006–2009 (2010)	Mixed methods: CHW database of 660 CHW and 5 focus groups with 35 CHW	Attrition rate 21 % over 30 months; Attrition was comparatively higher in younger age groups (25.9 % in 15–25 years group, 18.2 % in 26–45 years group and 16.5 % in ≥46 years group). Community acceptance of program was positively associated with retention.
2. Ahluwalia et al. (2010) Tanzania	n/a	Community-Based Reproductive Health Project (CBRHP)	Post-project assessment on a community-supported emergency transport systems, retention of village health workers (VHWs), and potential impact on maternal health	2001–2006 (2007)	Document review, community assessment survey, volunteer health workers survey	1. Community-financed transport system continued in 6 villages.2: CBRHP-trained village health workers have continued to work for more than 5 years and report on their activities during village meetings3. Significant improvement in women seeking prenatal care <20 weeks gestation, identification of pregnancy-related danger signs and improved maternal and infant outcomes.
3. Ajayi et al. (2014) Nigeria	n/a	Home-based management of malaria Nigeria	The extent to which the program continued, the prospects and challenges encountered such as with retention of community medicine distributors (CMDs) and the way forward.	2005–2007 (2010)	Focus group and key informant interviews	Utilization of CMDs was said to be high when the project started but dwindled after the researchers left the community. Majority of the caregivers sought care at other alternative care providers or used herbs.
4. Akogun et al. (2001) Nigeria	n/a	Community-based onchocerciasis treatment, Nigeria	Treatment coverage, acceptability and effectiveness of a program-designed (PD) and community-designed (CD) treatment strategies in 37 villages. Features of the community that may facilitate acceptable and sustainable community-directed treatment.	1995–1996	37 villages divided into two groups: PD versus CD. A total of 1744 people were interviewed about their experiences after two treatment cycles using household surveys, observation notes, in-depth interviews and focus group discussions.	1: Mean total coverage was 37.7 % with a range between 0 and 100 %. 5 villages had coverage rates above 60 % and dosage was correct in most cases (87.5 %). Most frequent reason for non-treatment was drug shortage (50 %) and being under-age (31.3 %).
5. Amazigo et al. (2007) 41 projects with African Program for Onchocerciasis control: Cameroon. Chad, Democratic Republic of Congo, Ethiopia, Malawi, Nigeria, Sudan, Tanzania, and Uganda	n/a	Community-directed treatment with ivermectin (CDTI)	Community ownership in long-term project sustainability defined as: “evidence of the ability of the community to own and manage CDTI; participation of community members and their leadership in decision-making; initiating and supporting CDTI implementation”.	1997 (2002–2003)	Study included randomly selected 41 projects in 10 countries (total 492 communities); methods included interviews, documents review, and observations. Quantitative and qualitative scores were used to obtain individual community scores and an overall sustainability score for each project graded on a scale of 0–4.	Of the 41 projects evaluated, 70 % scored satisfactorily to highly on sustainable at the community level.
6. Amo-Adjei (2013) Ghana	Sustainability framework	TB control, Ghana	Sustainability is conceptualized as the perceived and actual ability of the NTP of Ghana to continuously seek and attract resources to improve or maintain the existing levels of diagnosis and treatment of tuberculosis	2012	In-depth qualitative interviews with 19 stakeholders	The findings reveal two main strands of views about the sustainability of the current TB control programs: optimism and pessimism. The optimists revealed that the integration of TB into the generalized health system, integration of TB and HIV control services, the use of internally generated funds of health facilities, and a general improvement in socioeconomic conditions of the general population could provide positive pathways to sustainability. The pessimists on the other hand noted that the existing program was not likely to be sustainable so long as much of the operational funds were derived from external sources.
7. Aubel et al. (1996) Gambia	n/a	Community nutrition program	How can community nutrition programs be designed so as to favor sustainability involving the promotion of *futu kanya*, a traditional snack food made with millet, sugar and groundnut past as a dietary supplement for pregnant women.	1990 (1994)	Qualitative research using rapid assessment procedures involving in-depth interviews, semi-structured interviews, interviews with staff and document review.	Project was successful in terms of community involvement in the production and promotion of *futu kanya* which had a positive effect on the pregnant women who consumed it. *Futu kanya* was consumed at the recommended rate of 150 g daily.
8. Blanchet et al. (2014) Ghana	Sustainability framework based on diffusion of innovations; 5 components: health outcomes, service delivery, organizational capacity, viability, and community capacity; diffusion of innovations	An eye care program	Level of continuation of activities after the end of international funding in 11 of the 19 district hospitals. Measured by comparing the number of outputs per activity before and after the end of international funding (18 months after international funding ceased). It involved checking whether each eye care activity continued (i.e., outpatient consultation, cataract surgery, outreach, school health, and statistics) or was interrupted after the end of Swiss Red Cross funding	1996–2006 (2009)	Document reviews, in-depth interviews with 51 officers at the ministry of health, regional and district health authorities, district hospital managers, and health staff, Swiss Red Cross Officers, and community members.	School health screening was the least sustained activity after the end of international funding. In contrast, compared to the three other district activities, facility-based consultations were more likely to be routinized.
9. Blanchet et al. (2014) Somaliland	Sustainability framework	Physical rehabilitation network	Sustainability analysis process which involves 5 steps which are as follows: (i) establish a common understanding of the rehabilitation system in the local context, (ii) define system boundaries, (iii) develop a common vision of sustainability, (iv) select measurable sustainability indicators for the local system, and (v) collect baseline indicator data	2010 (2012)	In-depth interviews and observations using analytic narrative approach	In Somaliland, the small, centralized stakeholder network suffered a critical rupture between the system’s two main information brokers due to competing priorities and withdrawal of international support to one of these. Progress toward self-defined sustainability was limited.
10. Burlew et al. (2014) Nigeria	In-service training (IST) improvement framework; 6 broad themes: strengthening training institutions and systems, coordination of training, continuum of learning from pre-service to in-service, design and delivery of training, support for learning, and evaluation and improvement of training	PEPAR-funded in-service training (IST)	How implementing partners collaborate with each other in the coordination and delivery of HIV/AIDS related IST and to what extent training is effective, efficient, sustainable, and aligned with national priorities	2004 (2007–2012)	Stakeholder survey	Recommendations: improve collaboration and coordination among implementing partners; apply a more diverse and cost-effective set of training modalities; allocate funding specifically for the evaluation of the effectiveness of training; improve links between IST and both continuing professional development and pre-service education; require implementing partners to create sustainability plans to transition training from PEPFAR funding to other funding sources; and develop a training information management system
11. Eliason (1999) Cameroon	Freire’s conscientization theory	Life Abundant Program (LAP), church sponsored primary health care project	Continuation of LAP defined as: active which refers to functioning health promoters and financially viable village primary health centers (PHCs). Closed refers to the locking of the medicine box and cessation of health promoter activities	1980–1997 (1980–1997)	Evaluation of the first 16 LAP-associated primary health centers.	81 % of the 16-LAP’s were active 9 years after the first PHC opened, and 87.5 % after 17 years.
12. Fonck et al. (2001), Kenya	n/a	Decentralized antenatal syphilis screening program	Effectiveness of screening and testing seroreactive pregnant women and their partners using rapid plasma regain (RPR) and formulate recommendation for future implementation	1992 (1997–1998)	Evaluated data from 10 primary health care clinics, quality control data from referral labs, with information on costs.	96 % of all pregnant women attending the 20 clinics were screened for syphilis. RPR prevalence was 3.4 %
13. Ghiron et al. (2014) East Africa (Uganda and Kenya)	WHO’s 12 recommended tools for beginning the end in mind	Health of People and the Environment Approach	Beginning with the end in mind: planning pilot projects and other programmatic research for successful scaling up, which provide 12 recommendations to help ensure that a sustainable and scalable model is designed and tested, laying the groundwork for future success with scaling up	2003 (2011)	Participant observations, rural appraisal in project sites, key informant interviews, desk review of documents, in-depth interviews with 9 project team members and 13 project stakeholders	Previously funded PHE projects faced challenges of sustainability, and few reached beyond the confines of their original target communities. Rather than setting up parallel structures, the team has tried to work with, and within, existing personnel and systems.
14. Harpham et al. (2002) Tanzania	n/a	Urban Health Project	Capacity building and institutional strengthening	1990 (2000)	Key informant interviews, meetings with health management teams and health boards, document reviews, and surveys of users and community members	Project achieved improvements in capacity building and in structural and technical quality of care.
15. Humphries et al. (2011) Southern Africa (Botswana, Lesotho, Namibia, Southern Africa, Swaziland)	Johnson et al. (2004) sustainability planning model	NGO Training Institute to build capacity of NGO’s working to address HIV	Sustainability of (1) administrative structures, (2) innovation champions and leadership actions, (3) resources to support the innovation, (4) administrative policies and procedures, and (5) expertise to sustain the innovation and how these components were and were not incorporated into the NGOTI implementation	2004–2007 (2007)	Surveys and qualitative interviews to assess project outcomes: interviews with 44 internal stakeholders (trainers, managers, administrators, and technical advisors).	The NGOTI was able to develop the capacity of partner organizations in the area of AIDS NGO/CBO capacity building, as evidenced by the ability of these organizations to obtain additional funds to continue some of that work.
16. Hutchinson et al. (2010)	n/a	Obstetric case reviews, Benin	Adopting a multi-professional approach, engaging managers and key stakeholders, ensuring sufficient resources, and having an effective organizational structure with dynamic leadership	1998–2001	In-depth interviews	View that near-miss audits were valuable but that hospitals generally stopped performing them
17. Kachur et al. (1999) Kenya	n/a	Insecticide treatment material intervention	Whether community members had kept and continued to use their ITM, whether they had maintained and retreated them, and local attitudes toward ITM 3 years after the study ended	1990–1992 (1995 for follow-up)	Structured household questionnaire (QN) Focus groups	Participants identified malaria as a significant health problem in the community. Most noted that bednets were advantageous for preventing mosquito borne illness.
18. Katz et al. (2014) Benin, Kenya, Lesotho, Sierra Leone, and South Sudan	HIV/AIDS Programme Sustainability Analysis Tool (HAPSAT)	HIV program	Sustainability as characterized by the following: prioritization, efficiency improvement, and resource mobilization	2010–2012	Stakeholder interviews	The need to prioritize evidence-based interventions and apply efficiency measures is being accepted by countries. Five of the six countries in this study requested that the HAPSAT team prepare “prioritization” strategies. Three types of sustainability strategies were selected by stakeholders: prioritization, efficiency improvements and resource mobilization
19. Kuyini et al. (2011) Ghana	WHO model for community-based rehabilitation	Community-based rehabilitation program for people with disabilities	Sustainability depends on the balance of top-down versus bottom-up approaches to program implementation in a way that allows for meaningful grassroots participation, while at the same time attracting government support	1999–2000	Closed question survey with beneficiaries of disability service, local supervisors and social workers	Few CBR programs remained after funding ceased. Program officers had irregular contact with beneficiaries.
20. Le Gargasson et al. (2013) Congo	n/a	Public-private partnership to increase immunizations	Sustainability of routine immunization program performance and financing.	2002–2010	Review of published and gray literature, and interviews with stakeholders in Congo to assess allocation of funds	DTP3 coverage increased from 2002 (38 %) to 2007 (72 %) but had decreased to a level below 70 % in 2008 (68 %) and 2010 (63 %). The overall funding for vaccines increased from US$5.4 million in 2006 to US$30.5 million in 2010 (mostly from GAVI support for new vaccines). However, during the same period, the funding from national (government) and international (GAVI and other donors) sources for routine immunizationservices (except vaccines) decreased from US$36.4 million to US$24.4 million. This drop in overall funding (33 %) primarily affected surveillance, transport, and cold-chain equipment.
21. Lindblade et al. (2004) Kenya	n/a	Malaria prevention	Continued surveillance of adherence	Phase 1: 1997–2000 and Phase 2: 1999–2002 (2002)	Community randomized control trial	The public health benefits of insecticide-treated bednets were sustained for up to 6 years. There is no evidence that bednet use from birth increases all-cause mortality in older children in an area of intense perennial transmission of malaria
22. Mbanefo et al. (2010) Nigeria	n/a	Community-directed treatment program for onchocerciasis (CDTI)	9 sustainability indicators: 5 of these indicators assessed the routine project activities and processes: planning, leadership, monitoring and supervision, Mectizan (ivermectin) supply and distribution, and training/health education/sensitization/advocacy/mobilization (TRHSAM). Three indicators assessed resources available to projects: financing human resources, and transport and material resources. The output indicator assessed the therapeutic coverage; 65 % being the threshold required to achieve control within 15 years	1997 (2008–2009)	Structured questionnaires and focus group discussions	Coverage: 90 % (adequate by WHO 65 % standard) Planning: efficient but depended greatly on external resources and worker resilience Leadership: rested on community leaders who are unable to accept financial responsibility Finance: no organized system Human resources: lack of motivation Transport: logistics for transport of medication is not fully assumed by the communities. Overall, program can be sustained and disease eradicated, but efforts need to be intensified and strategies improved.
23. Mutale et al. (2015) Zambia	Sytems thinking-guided analysis framework	The BHOMA intervention (Better Health Outcome Through Mentorship and Assessment)	Improvement in service quality leading to increased service demand from the community	2011–2014 (2011–2012)	In-depth interview guides and focus group discussions	In the short term there was increased demand for services but the health worker capacity was not severely affected. However, from a systems perspective, unintended consequences also occurred during the implementation of the BHOMA
24. Osawa et al. (2010) Zimbabwe	Bennet et al. (2002) model of motivational processes	Community home-based HIV care program	Health worker motivation and satisfaction with workplace environment	1992 (2006)	Self-administered structure questionnaire for health workers	Motivation of workers linked with perception of family and community environment and perception toward organizational characteristics, specially managerial support, like attention from a manager, clear instruction, and goals, had an impact to CFs motivational outcome
25. Rashed et al. (1997) Benin	Participatory conceptual framework on sustainability	Participatory research program to develop local capacity to produce and market bednets	3 criteria:1. Participation of local human resources (program would be sustainable with existing local resources) 2. Self-financing 3. Appropriate action based on comprehensive knowledge of a local setting with enterprise adapted to local customs	1992–1994	Prevalence survey to establish rates of bednet utilization	Use of bednets increased, as well as sense of community pride for being able to address the problem. The mobilization of local human capacity, local financing of the purchase of bed nets in the rural setting and the participation of key local persons who produced tools adapted to the population, resulted in the development of an easy to produce and acceptable bed net.
26. Rassachert et al. (2014) Mozambique	Conceptual framework on sustainability based on previous literature	Community-based delivery of anti-retroviral—a process where patients take an active role in ART provision in the community	5 main components of sustainability: (1) design and implementation processes, (2) organizational capacity, (3) community embeddedness, (4) enabling environment, and (5) context	2008–2012 (2012)	In-depth interviews with: patients, nurses, lay counselors, health authorities, program implementers	The community embeddedness of the model, together with patient empowerment, high acceptability and progressive MoH involvement strongly favor the future sustainability of the CAG model. The high dependency on external resources for the model’s daily management, however, can potentially jeopardize its sustainability.
27. Rosenberg et al. (2008) Botswana, Lesotho, Namibia, South Africa, and Swaziland	n/a	Community-based orphans and children project	The continuation of benefits and activities achieved during the project after donors’ funding has ceased	1999 (2003–2006)	Site visits include document review and interviews with organization leaders, staff, partners from other organizations, and recipients of services. Focus groups with constituents are also conducted	For eight of the nine projects, evaluations provided evidence of the importance of the government partnership for sustainability. Government collaboration was important in projects designed to help families access government grants, initiate community-based solutions, and advocate for OVC rights through legislation. Government partnerships were also critical to the sustainability of two projects involved in placing children in foster care, but these showed signs of tension with government partners other factors included:organization, NGOs and donors should develop strong partnerships with local and national funding agencies.
28. Rourra et al. (2009) Tanzania	Socio-ecological framework	Community-based cohort study for ART	Factors underlying attendance at ART clinics	2006	Semi-structured interviews with clients, service providers	Personal motivation and self-efficacy contribute to program retention, along with perceived health benefits and disease severity. However, these determinants are influenced by others’ opinions and beliefs in the community, and constrained by programmatic and structural barriers.
29. Sarriot et al. (2015) Rwanda	Sustainability framework	Integrated community-case management of malaria, pneumonia and diarrhea	The sustainability framework examines the maintenance of positive health outcomes, or their continued improvement, through social and institutional arrangements between stakeholders	2011	Secondary data analysis and causal loop diagram	Financial, political and technical scenarios carry high probability for threatening the sustainability through: (1) reduction in performance-based financing resources, (2) political shocks and erosion of political commitment for community health, and (3) insufficient progress in resolving district health systems—“building blocks”—performance gaps
30. Sebotsa et al. (2007) Lesotho	n/a	Salt iodization program evaluation	WHO criteria for sustainable elimination of iodine-deficiency disorders such as: existence of an effective, functional national body responsible to the government for the national program for the elimination of iodine-deficiency disorders; appointment of a responsible executive officer for the iodine-deficiency disorders elimination program; legislation or regulations on universal salt iodization; cooperation from the salt industry in maintenance of quality control	2000 (2002)	Chemical analysis of urine samples and in-depth interviews with the chairperson of the iodinedeficiency disorders control program to assess indicators of sustainability.	Iodine deficiency was eliminated as a public health problem, as rates of deficiency were less than 10 %. But this elimination is not sustainable. Effective regular monitoring of salt iodine content at all levels, with special attention to iodization of coarse salt, is recommended, together with periodic evaluation of the iodization program.
31. Sharma et al. (2013) Kenya, Zambia, and Nigeria	Clinical Assessment for Systems Strengthening (ClASS) framework	Evaluate the role of Clinical Assessment for Systems Strengthening in building local partner capacity for HIV care	Building capacity of local partners to endure and adapt to changing financial and policy environments	2010–2011 (2011–2012)	Individual and group interviews with key stakeholders	Implementing the ClASS framework led to changes in policy and practice, continuous quality improvement initiatives, and consolidation of partnerships, all of which improved internal operations. CIASS had become part of the organization’s capacity building.
32. Somasse (2013) Burkina Faso	Community-based management of acute malnutrition (CMAM) of the Belgian Red Cross	Community-based management of acute malnutrition	Which activities of the program the community or the health system could continue to conduct even if the program funding stopped	2006 (2010)	Document analysis of program reports, individual interviews and focus groups	Recovery rates were about 87 %. The health district medical offices agreed that the program was effective and helped the communities to understand the problem of malnutrition and helped increase the use of antenatal care and health services
33. Swain et al. (2014) Rwanda	n/a	Collaboration between expatriate humanitarian cardiac surgery program and the National Health Foundations	Strengthen care on three levels: (1) expanding local capacity for cardiac surgery, (2) reinforcing registry-based secondary prophylaxis, and (3) enhancing treatment of streptococcal infections	2008–2013	Interviews with key personnel and review of administrative records; surgical cases completed and the resulting outcomes	86 patients have been seen with 123 valve replacements. Since 2008, the program is now treating patients with more complex diseases.
34. Teguete et al. (2012) Mali	n/a	Visual cervical screening	Improving cervical cancer control provision by health services and sustaining visual screening as part of routine health care in Bamako and surrounding areas after the completion of the research project	2004–2009	Routine visual screening and treatment services	Finding suggest that it is feasible to sustain good quality visual screening services in a low-income country such as Mali by maintaining and using the resources originally provided for a research project and by utilizing the resources available in government health services.
35. Torpey et al. (2011) Zambia	Sustainability conceptual framework	HIV services	Service sustainability, a complex concept that can be classified into four elements: technical, programmatic, social, and financial sustainability	2004–2009	Quality assurance and quality checklists through structured set of data collection tools, involving checklists, interviews by healthcare workers and patient record reviews.	Achieving operational sustainability in a resource-limited setting is practical and feasible. Developing and institutionalizing a quality assurance/quality improvement system is the basis of attaining graduation and sustainability of services.
36. Maticka-Tyndale et al. (2010) Kenya		Primary School Action for Better Health (PSABH) AIDS prevention	Program delivery and impacts of curriculum on student behavior	2001–2004	Surveys with teachers and students, focus groups with students and in-depth interviews with teachers.	Teachers continued to deliver program components three years after they were trained. Gains demonstrated in pupil knowledge, attitudes and risk-reducing sexual behaviors after one-and-a-half years of program implementation were replicated in the third year of the intervention with additional gains in attitudes related to condoms and girls’ reported use of condoms.
37. Vamos et al. (2014) Zambia	The “train the trainer” model	HIV risk reduction behavioral intervention for HIV seropositive and serodiscordant couples	Sustainability defined as retention of interventionists and clinic staff.	2008–2013	Data were collected from CHC sites on current employment status of participating CHC staff, and the reasons for discontinuing employment (e.g., transfer, study leave, retirement). The number of cohorts conducted by each interventionist was recorded, in addition to the continued provision of the intervention post-study completion at the CHC	High levels of clinic burden were identified; however, no increase in perceived clinic burden or staff burnout was associated with providing the intervention. The intervention was sustained at the majority of CHCs and also adopted at additional clinics.
38. Walsh et al. (2012) Zambia	Framework for the assessment of sustainable community-based organizations	Multi-country AIDS program	A contribution to the development of conditions enabling individuals, communities and local organizations to express their potential, improve local functionality, develop mutual relationships of support and accountability, and decrease dependency on insecure resources (financial, human, technical, informational) in order for local stakeholders to negotiate their respective roles in the pursuit of health and development, beyond a project intervention	2003–2008 (2010–2011)	In-depth interviews with district level representatives from community-based organizations	Funding opportunities for CBOs in Mumbwa in 2010 were scarce. Health services: While all CBOs were functioning in 2010, most reported reductions in service provision. Home visits had reduced due to a shortage of food to bring to people living with HIV/AIDS and scarcity of funding for transport, which reduced anti-retroviral treatment adherence support and transport of patients to clinics. Organizational capacity and viability: Sustainability had been promoted during MAP through funding Income Generating Activities. However, there was a lack of infrastructure and training to make these sustainable. Links between health facilities and communities improved over time, however volunteers’ skills levels had reduced.
39. Wandeler et al. (2012) Zimbabwe, Mozambique, and Lesotho	n/a	ART retention program	Examined the importance of no follow-up after initiation of ART as well as mortality and loss to follow-up (LTFU) over three years of ART	2005–2010	Patient records from day 1 of ART treatment through follow-up; random quality checks for sites	A total of 9271 patients started ART during the study period. Overall 449 patients (5.8 %) were not seen after the ART initiation visit. Over 9575 person-years of follow-up 1319 patients (18.1 %) of the 7276 patients with at least one follow-up visit were LTFU and 698 patients (9.6 %) died. The crude mortality rate was 7.3 (95 % CI 6.8–7.9) deaths per 100 person-years.
40. Wilson et al. (2014) Ghana	n/a	Continuous positive airway pressure (CPAP) trial	The extent to which the skills and equipment necessary for CPAP use have been maintained.	2011 (2013)	Assessment of CPAP skills in first-generation and second-generation nurses who underwent training and equipment inventory	First-generation trainees scored significantly higher than second-generation trainees on both skills and knowledge assessments. Appropriate + technical support and training must be ensured to address equipment maintenance. Protocolization of the training program, in conjunction with skills and knowledge assessment, may improve acquisition and retention among second- and future-generation trainees.
41. Zulig et al. (2014) Tanzania	Weiner’s theory of organizational readiness to change	Cancer registry program	Weiner’s theory of organizational readiness to change provided the conceptual model was used to define sustainability. The key tenet is that organizational readiness is a multi-level, multi-faceted construct comprised of both organizational members’ shared resolve to implement a change	2013	Interviews with administrative department heads and clinical stakeholders	Nearly half (45 %) of participants discussed change commitment, stating that the cancer registry would be of benefit to them and that they were committed to it. However, change efficacy was low—participants were not confident in their shared ability to sustain the registry. Most participants (73 %) discussed the importance of resource availability and administration support.

*n/a* not available

### Theory or framework used

Twenty three of the 41 articles reviewed discussed framing the sustainability in terms of a theory or conceptual framework. The most common framework utilized was the sustainability framework, which was discussed in four of the studies. Three studies used the socio-ecological model of behavior, which was the second most frequent framework. Eight of the studies discussed a framework for the research that could not be classified as a traditional theory. These included WHO recommendations for sustainability, sustainability analysis tools, and improvement frameworks.

### Type of methods used

Majority of studies reviewed utilized qualitative evaluation methods, most commonly in-depth or semi-structured interviews (*n* = 17). Many of the qualitative inquiries also used additional methods such as document review and observation for triangulation. Mixed methods studies comprised one quarter of the reviewed papers (*n* = 10). Some of the studies used a structured quantitative questionnaire along with either individual interviews, focus groups, key information interviews, or a combination. Thirteen studies explicitly evaluated evidence-based interventions with regard to sustainability, and three of the twelve were randomized control trial. Other methods of evaluation included use of laboratory tests, reviewing clinic processes (e.g., referral rate, staff retention, staff burnout, etc.), and patient record review.

### Timeframe assessed

We coded studies for the last post-implementation timeframe reported. Most studies (*n* = 22) occurred 12 months or more past the initial implementation. Eighteen (44 %) reported outcomes at less than 12 months post-implementation, three (7.3 %) at 12 months, and nine (22 %) between 12 and 36 months post-implementation. Ten studies (24.4 %) were evaluated more than 3 years after implementation. While studies have suggested the need to consider sustainability elements over time, only one study conducted in Kenya examined sustainability gains made with their intervention at two time points.

### Health outcomes reported

The health outcomes reported in the studies were diverse. Nineteen out of 41 studies (46 %) reported sustainability outcomes focused on communicable diseases, with HIV and AIDS (12/19, 63 %) represented in majority of the studies, followed by malaria (5/19, 26 %). Six out of the 41 studies (14.6 %) focused on non-communicable diseases, four studies (9.8 %) focused on reproductive health as well as health promotion (9.8 %) in sub-Saharan Africa, while three studies (7.3 %) focused on neglected tropical diseases, two studies on rehabilitation services, and the remainder on quality improvement of health care delivery, eye care, and the sustainability of immunization programs in the region.

### Sustainability definition and outcomes reported

Although all the studies focused on aspects of sustainability, only 21 out of 41 (51.2 %) studies had clear definitions of sustainability. The remaining studies described sustainability using terms or factors such as program continuation/maintenance, program effectiveness, functioning, routinization, and capacity building, as well as retention of workers and community ownership of project. Among the studies with clear description on sustainability, the definitions cited were based on sustainability as defined from previous literature such as the work of Shediac-Rizkallah and Bone [21], Sarriot et al. [29], and Johnson et al. [30] and the sustainability planning model, as well as the existing definitions from the World Health Organization. In terms of sustainability outcomes, majority (whether they defined sustainability or not) reported outcomes related to the continuation of the program (46.3 %) such as whether activities continued or were interrupted following the end of funding. Others focused on ownership of project (24.4 %) whether at the community level or structural level, effectiveness (7.3 %), capacity building (7.3 %), retention of workers (7.3 %), and routine use of interventions (5 %) following initial implementation. Only one study focused on quality improvement of the intervention [31]. Also, among the thirteen evidence-based interventions reviewed, six reported data on the extent to which patient-level or individual factors were sustained following the end of the implementation, while six assessed sustainability at the provider level. Studies focused on sustainability at the provider level examined retention of health care workers and the challenging work conditions they experience at the end of a project. One study examined the sustainability of an equipment used during a clinical trial [32], while another evaluated sustainment at the community level [33].

### Narrative synthesis of findings

The findings were further grouped into three main themes presented in Table 2 which describe either facilitators or barriers toward sustaining health interventions in sub-Saharan Africa.

**Table 2:** Summary of factors considered as facilitators and barriers toward sustainability of health interventions in sub-Saharan Africa

Facilitators	Examples	Barriers	Examples
Community ownership	1. The highly centralized structure of the social network potential to help rapidly diffuse information between actors [34] 2. Community mobilization [66] 3. (a) Community involvement in meetings; (b) collective ownership; (c) inputs from professional in health system to include local economic concepts and values [67] 4. Community ownership, responsibility, and participation [33, 39, 44] 5. Regular dialogue with community; community ownership [40] 6. Engaging in participatory process with key stakeholders [41] 7. Builds on social and cultural values [38, 63] 8. Creating strong social links and networks with members; social support [38, 43] 9. Resource flow between members of social networks, [38, 66]	Weak health systems	1. Volunteer health workers need refresher training and proper supervision [66] 2. Limitations with assessment of sustainability over time [67, 68] 3. Severe shortage of drugs [67, 68] 4. Weaknesses with formal health systems with timing of distribution of medical services [33] 5. Lack of community-managed monitoring and supervision system [39] 6. Poor assessments [69] 7. Lack of collaboration and access to data 8. Lack of provider integrity [40] 9. Lack of comparable baseline data [17] 10. Lack of rigorous models evaluating sustainability of community health worker programs [61] 11. Lack of monitoring and reporting; no central database for recording [47] 12. (a) Need updated risk management, (b) lack of structure for decision-making, (c) need to improve referral and dissemination of results [50] 13. Lack of Ministry of Health recommendations on how to integrate the program activities into the daily planning and strong strategic plan [42] 14. (a) Fragile and understaffed health systems; b) lack of access to viral load monitoring [52] 15. Lack of disease registries; paper-based patient records [55]
Working within existing resources	1. Institutionalization and integrating within existing political and economic resources [66] 2. The use of a respected traditional authority (i.e., village heads) [33, 67] 3. Adaptation to cultural norms and values [33, 39, 67]; tailoring innovation to sociocultural and institutional settings [41] 4. Building on existing social units and roles such as traditional communicators, traditional birth attendants, and community management committees [39] 5. Consideration of the individual parts (e.g., activities) of a health program as it is to consider the program as a whole [34] 6. Continued dialogue with community members [41] 7. Building on pre-existing capacity of community-based organizations to organize themselves [57]	Lack of financial leadership	1. Lack of remuneration for caregivers [70] 2. (a) Lack of long-term planning [61, 71] 3. Reliance on external funds [40, 71, 72] 4. Lack of funds [43–45, 69, 17] 5. Financial disbursements [43, 45] 6. Availability of resources [43, 73–76] 7. Lack of motivation and incentives [70] 8. (a) Absence of functional financial institution to receive and transfer funds to sub-national levels; (b) incentives did not benefit staff; (c) lack of budget and accounting organization; (d) limited contribution of domestic resources [70] 9. (a) Constraints due to financing and vertical selection of programs; (b) free distribution approach weakens health system [35] 10. Conflict over fund allocations and patient difficulty paying fees [38] 11. Inability to guarantee continuity of future resources [72] 12. Lack of communication about funding termination [57] 13. Lack of medical equipment and uncertainty about securing future funds for equipment [32]
Community buy-in through volunteerism	1. Satisfaction of being able to contribute to community well-being [70] 2. Incentives/recognition by cardinal staff and community leaders [68, 70] 3. Supportive community environment [68] 4. Perceived benefit of intervention [33, 39] 5. Indirect benefits including happiness serving their people [33] 6. Support from key community leaders; motivation, training and supervision of community actors [39] 7. Strong community support [73] 8. Community acceptance [17] 9. Include stakeholders in discussion and planning [50] 10. Community volunteers perceived their role as close to that of a health worker in the community [42]	Health care worker shortage	1. Weak sense of social responsibility [70] 2. Staff workload; prolonged crisis in staffing [44] 3. Longer wait times due to overworked staff; staff working longer hours for less pay [31] 4. Volume of demand, equipment and staff shortages, inadequate management, limited supervision, high turnover, [77] 5. Health worker training in light of “brain drain” [48] 6. (a) High workload and patient volume, (b) limited resources and space [51] 7. Lack of staff [55]
Sound infrastructure	1. Community leadership support and administrative structures to foster supportive environment, efficiency, and commitment [33, 40, 45, 17, 57, 74, 76] 2. Resource contribution; resources to support innovations [33, 45, 46, 75, 51] 3. Record keeping and reporting; improved monitoring and reporting, quality improvement cycles initiated [33, 50] 4. Development and accreditation of standard training, education, and evaluation materials along with training and oversight [41, 45, 48, 63, 32] 5. Integrity in money management [40] 6. Promote learning and disseminate information [41] 7. Establishment of health facility board; development of community-based health care implementers; the community health boards monitored revenue collection and expenditure of cost-sharing funds; decentralized approach of services integral to health care with national supervision [44] 8. Good and well trained health care workers; consistent delivery of services [31, 37, 52] 9. Strategies based on key informants; (b) participation of non-governmental groups to provide experience with operationalization of a project [35] 10. Integration of staff, communication, political support, leadership, participation;[43]; integration of academic, government, and faith based organizations [77] 11. Strong political will to promote health; dynamic community health governance; systems approach to sustainability [61] 12. (a) existence of an effective, functional national body responsible to the government for the national health programs [47] 13. Several point-of-care services, with an in-built referral pathway for diagnosis and treatment [56] 14. (a) Enforcing use of standard guidelines; (b) staff training, mentorship, and technical support; (c) strengthening ministry’s supply and logistics for procuring and maintaining services; (d) quality assurance/quality improvement system provided basis for continuous assessment and monitoring of services [49] 15. (a) Social cash transfer scheme at national level; (b) coordination between health resources at district and community levels [57] 16. Capacity building through skill building [32] 17. (a) Open communication; (b) support from hospital administrators; (c) international partnerships [55]	Lack of education and awareness	1. Shortcomings in the knowledge and attitudes of members of the community concerning maternal health and nutrition [37, 39] 2. Weaknesses in medical skills training; lack of training for community engagement [44] 3. Lack of knowledge of disease risk or transmission [73] 4. Health education and community empowerment [36] 5. Social norms and misconceptions [38] 6. Insufficient public education and lack of awareness [47] 7. Lack of awareness and advocacy, need to mobilize resources [56] 8. Minimal community awareness [57] 9. Low literacy [52] 10. Poor knowledge retention [32]

#### Facilitators

##### Community ownership

Community ownership and mobilization were recognized by many of the reviewed studies as crucial facilitators for intervention sustainability, both early on and after intervention implementation. Involvement of stakeholders and providing them with a sense of ownership in intervention proved beneficial for a variety of reasons. In Somaliland, a physical rehabilitation network evaluation found that a centralized social structure within the community allowed for rapid diffusion of information between various actors in the intervention, which would aid tremendously in the context of an emergency [34]. In Zambia, commitment and ownership positively affected a program designed to address quality improvement in health service delivery [31]. Another study by Rashed et al. [35] that investigated the impact of insecticide-treated bednets used to prevent malaria found that community members felt a great sense of pride in participating in the program. The stakeholders felt that they were able to do something themselves that led to disease reduction, which increased their feelings of ownership and motivation to continue the program. For certain interventions, community involvement reduced stigma surrounding the disease [36, 37]. Involvement of key stakeholders in implementing interventions and recruiting community members can ensure that the appropriate social norms are addressed during recruitment and program awareness. For instance, in the context of HIV/AIDS, social stigma is a barrier that prohibits participation in prevention and treatment programs and can hinder the sustainability of programs targeting HIV. Osawa et al. [37] found that having community care facilitators for an anti-retroviral treatment program helped reduce community stigma surrounding HIV and led to better program retention. Perceived social support from community and family members is also important for intervention sustainability, especially for HIV/AIDS treatment programs [38].

##### Working within existing resources

Other facilitators identified in only a handful of studies were building off of, and within, existing community resources. This serves as a unique aspect of intervention implementation and sustainability as working within existing resources ensures that a framework component of the intervention already exists and can continue to exist in the absence of external funding and assistance. For instance, an assessment of a community nutrition program for pregnant women found that building on existing social units and roles, such as birth attendants and community leaders, enabled the sustainability of efforts to produce and promote a nutritional supplement to improve women’s nutrition during pregnancy. Additionally, the researchers noted that interventions that are compatible with social norms and values such as the use of traditional songs or plays can contribute to sustainability in the long run [39]. Eliason [40] incorporated unique aspects of the community in their intervention as well. In order to improve primary care, the authors utilized churches in the community as the intervention setting, thus incorporating the intervention directly into an existing and thriving part of the community. Through establishment of project leadership and direct dialogue with community members, these primary care centers were sustained upwards of 9 years. Eliason [40] noted that the church was instrumental given its philosophy of being a center of wholeness with the responsibility to minister to the underprivileged and suffering in the community. More recently, Ghiron et al. [41] implemented an environmental approach to improving health through continued dialogue with community members and social/cultural tailoring. Furthermore, instead of creating a parallel structure, the team aimed to work within the existing personnel and systems. Another unique factor that facilitated sustainability was flexibility and local adaptation of interventions to unique contexts. Amazigo and colleagues [33] observed that helpful adaptations to interventions are a sign of the sound leadership needed to sustain local interventions. Blanchet et al. [34] suggested that disentangling projects into distinct activities allows sustainability to be achieved and helps identify which activities are likely to be maintained and which activities are more likely to stop.

##### Community buy-in through volunteerism

An intervention to treat acute malnutrition in children in Burkina Faso demonstrated the importance of volunteerism in intervention sustainability [42]. Somassé et al. [42] found that community volunteers perceived their role in the program as similar to other community health workers (e.g., nurses, physicians) and thus showed good ownership of the intervention and were ready to take on various responsibilities to continue the intervention. The importance of community ownership and intervention compatibility with community norms and values was noted in several of the reviewed studies that addressed a wide variety of health interventions, ranging from community nutrition projects to eye care programs. Incorporating community values and integrating key stakeholders in the development and implementation of interventions increases the likelihood of sustainability, as community members feel more ownership and involvement than they would in a program that does not align with their cultural and social norms. Rassachert et al. [43] built upon this, discussing the importance of program flexibility in order to adapt to different cultural norms when appropriate. Flexibility of health programs facilitated staff integration and communication and created an enabling environment in which the workers accepted the intervention. Furthermore, the ability to tailor programs based on specific community needs while involving key stakeholders can help initiate meaningful participation, despite the fact that this can have significant time implications [41].

##### Sound infrastructure

Another theme that emerged from the articles reviewed was the importance of developing an infrastructure for interventions and delegating responsibilities for intervention maintenance. Studies noted that a facilitating factor to intervention sustainability was the development of a community advisory board, health facility board, or administrative structure [44, 45]. Le Gargasson et al. [46] discussed that a centralized civil society organization enabled the maintenance of a public-private partnership to increase immunizations because this organization was pivotal in securing program funding. Similarly, in evaluating a salt ionization, Sebotsa et al. [47] found that the presence of a functional national body responsible for governing the ionization process, appointment of reliable executives, and legislative regulations on the ionization process were key facilitators to program sustainment. Indeed, the importance of reliable leaders who take control of the tasks necessary to sustain a health program is a cornerstone for intervention maintenance. Also, the need for better monitoring and program assessment was described as a factor likely to influence the sustainability of interventions. The continued assessment and reassessment of these interventions is necessary to ensure that protocols are being followed and that the interventions are being implemented in a culturally and socially appropriate way. Furthermore, centralized databases for this information facilitates the sustainment of interventions over time as they pass to new management and new leaders, to ensure that original and/or effective protocols are being followed.

#### Barriers

##### Weak health systems

Broader social and ecological conditions as well as societal upheavals were barriers that influenced the sustainment of community-based interventions in sub-Saharan Africa. For instance, in Mozambique, district health authorities suggested that weak health systems and poor health coverage limits the sustainability of most community-based health programs in the region [43]. A cardiac surgery program in Rwanda faced tremendous difficulties due to the aftermath of the genocide, in which most health care professionals fled the country [48]. In Zambia, Torpey et al. [49] suggested that the fragile state of the health care system creates a challenging environment that affects technical, programmatic, and financial efforts of the Ministry of Health, Zambia, to contribute toward building long-term sustainable HIV interventions. The political conditions of certain countries posed unique challenges with respect to intervention implementation and sustainability.

##### Lack of financial leadership

Furthermore, as previously mentioned, the lack of financial leadership and accounting organization hindered the progress made by interventions. One of the challenges with sustainability is the lack of funds available for a long term. Particularly for public health interventions, the lack of financial resources results in the use of inadequate equipment to perform necessary health interventions. For instance, La Gargasson et al. [46] noted that primary challenges to introducing new vaccines were insufficient funds for proper cold-chain processes and distribution costs. Rashed et al. [35] noted that financial constraints led to the cutting of potentially beneficial health interventions due to the lack of immediate benefits. The financial constraints faced in many low-resource settings force difficult decisions to be made about terminating potentially life-saving health programs in favor of others with more instantaneous results. Many of the beneficiaries of these health programs cannot afford to pay for services.

##### Leadership delegation and consistent workforce

Even at the community level, the need to delegate responsibilities to various community workers is key for sustainment. Mbanefo et al. [36] found that a major downfall of a community program for treating onchocerciasis was there was no structured mechanism for transferring financial responsibility for the intervention to the community. La Gargasson et al. [46] corroborated these results, demonstrating that the absence of functional financial institutions at both national and sub-national levels hinders intervention sustainability as there is no structured mechanism for allocating funds. Additionally, a lack of local record keeping poses challenges to sustainability because information regarding the amount of resources needed and previously used is not available for future use. Overall, better monitoring and program assessment is needed to sustain interventions. Sharma et al. [50] noted that prominent barriers to sustaining their program were the need to update risk management and protocol, a lack of structure for decision-making, and the need to improve disseminations. The effect of financial constraints on health workers is also a noted challenge. Many health workers participating in these interventions are either paid little or nothing for their contribution, leading to high rates of attrition and turnover. Additionally, high patient load and staff burnout were common barriers noted, primarily because programs could not afford to hire more staff to treat patients. Often staff were overworked which led to inadequate provision of services. Furthermore, staff in some cases, were mistreated, making retention difficult [44]. More generally, limited medical resources and the lack of space contribute to health care worker burn out and difficulty with intervention sustainability [43, 51, 52]. In many cases, health interventions lacked cohesiveness and coordination with political and social groups [53] leading to a lack of financial integrity [40] and instability with long-term financing [34]. Indirect costs associated with interventions such as patient and health worker transportation and far distances to clinics were also barriers to intervention sustainability [44, 38]. Health care workers often bare the financial burden of traveling to beneficiaries themselves, severely limiting the financial incentives they gain from working with the program.

##### Health care worker shortage

Another notable barrier to sustainability is the fact that many countries in sub-Saharan Africa already crippled by severe health care worker shortage. In some instances, the social and political climate in certain countries further weakens the already fragile health care systems, thus limiting capacity building and future sustainability. In one study, sustainability was difficult to achieve due to the overarching perspective that research efforts are seen as extraneous to medical treatment [54]. Osawa et al. [37] also noted that discordance between policy and community goals hindered the success and sustainment of an intervention. For instance, the lack of policy related to intervention evaluation and data dissemination conflicted with information presented to the intervention staff about the importance of evaluation. Humphries et al. [45] discussed this idea in the context of a program working to address HIV. Despite strong administrative structures, innovation champions, and leadership action, there was a lack of successful development and accreditation of training materials. Although the program itself had a strong infrastructure, the national policies related to development of accredited training materials was not consistent with the program goals and may have contributed to the lack of program ownership displayed by the community.

##### Lack of education and awareness

Nonetheless, the lack of community awareness or education regarding the health issue also impeded successful implementation and intervention sustainability [55]. Both Sharma et al. [50] and Walsh et al. [56] found that lack of program awareness among community members limited enrollment and retention in HIV treatment programs. Moreover, Zulig et al. [54] evaluated the effectiveness of a cancer registry and found that both lack of awareness of its existence the ambiguity of the program purpose hindered intervention progress. Similarly, a prenatal care intervention reported that women attending prenatal care late, either due to lack of awareness of services or pregnancy, was a barrier to sustainability [57]. Several studies also noted that lack of patient education specific to disease, general health education, and inefficient public education systems contributed to difficulties with sustainability [43, 47, 36]. Shortcomings in knowledge and attitudes toward specific health interventions among community members make ensuring the development of a community-managed monitoring system difficult, thus impacting overall intervention maintenance. Community provision of support must occur in a context in which the community is accepting of the intervention and believes that the benefits outweigh the cost [39]. In some cases, community fear of a disease, such as HIV/AIDS, is enough to prevent program implementation and maintenance [37]. HIV/AIDS is unique because of the associated stigma across the continent; however, stigma and community misconception of the disease prohibit individuals and communities from even participating in HIV interventions [38].

## Discussion

### Have gaps in knowledge been addressed in sub-Saharan Africa?

Since the first systematic review published in 2012 on the sustainability of new programs and interventions [22], sub-Saharan Africa has amassed a small but growing number of studies from 7 to 41, focused on the sustainment of health interventions implemented in the region. Most of the studies largely focused on answering the question of *what is sustained* after the initial implementation ends, with very limited studies focused on *how* or *by whom*, *how much*, and *by when* over several time periods [16, 21]. Indeed, a commonly held view is the idea that sustainability occurs at the end of the implementation process with post-project assessments used to evaluate whether intervention outcomes can be sustained. Not surprisingly, the findings of many of these studies indicated that interventions in sub-Saharan Africa are difficult to continue effectively and with fidelity to practice. This is because many studies did not necessarily plan for sustainability at the outset of implementation. Instead, across the studies reviewed, sustainability seemed to be an “added-on” element after an intervention has been designed, funded, and implemented.

The literature to a large extent also echo the findings of Stirman et al., specifically the fact that the literature on sustainability in SSA context is *“fragmented and underdeveloped”* [22]. In addition, although studies do provide some operational definition of sustainability guided by published literature or model or concept, the research itself remains largely descriptive and retrospective with few comprehensive or methodological rigor applied in the studies [22]. In studies with no definition, what is meant by sustainability is vague with the “sustainability” concept not clearly defined or consistently applied. The ability to interpret research findings or make cross-study comparisons about the extent to which interventions are sustained over time remains difficult to assess due to variety in terminologies and sustainability outcomes identified in available studies.

In addition to concerns with definitions, the review by Stirman et al. [22] also strongly recommended that there needed to be improvements in methods employed to characterize intervention sustainability and its influences, yet this area remains problematic in sub-Saharan Africa. Majority of the studies had limited descriptions of methods, few details of analysis, nonexistent benchmarks to guide researchers in efforts to identify the extent to which interventions were continued as implemented, and little or no recommendations on how to move sustainability research forward. Also, with the exception of few studies [33, 36, 43, 45, 61], there are still limited data on the processes associated with sustainability. Despite the recommendation to examine what is currently known about sustainability over time so as to capture variations over time [16], only one study conducted in Kenya assessed sustainability two time points over several years [58, 59]. Also, none of the interventions were developed specifically to refine hypothesis on sustainability drivers or promote the sustainability of effective practices. Instead, sustainability in many cases, drifted in favor of addressing resources needed to continue implementation with few focused on sustaining health outcomes over time. These limitations makes it exceedingly difficult to report the methodological rigor that might have been applied to the studies reviewed and contributed to the difficulties associated with understanding the intervention characteristics or practices that matter for long-term sustainability of health interventions implemented in the region.

### The importance of context

Sustaining interventions in the region anytime from 6 months following the end of a project or up to 9 years as in the case of Cameroon is of major public health significance. We found that facilitators such as community ownership of the interventions alongside building on existing unique resources such as churches and community health workers not only are important for sustaining interventions in sub-Saharan Africa but are also influenced by barriers such as the effect of financial constraints or the shortage of health care workers. Among the notable findings observed, allowing communities to optimize interventions according to their needs within the context of an overarching framework or promoting a process that is firmly embedded in traditional and cultural norms or values of local communities with support for community leaders and the use of local resources enhances the sustainability of interventions in the region [31, 33].

Nevertheless, individual or community strengths cannot overcome some of the weaknesses further up at the organizational or institutional level. The sustained use of interventions in the region also seemed to be influenced by the social conditions unique to the region whether it is with societal turmoil or shortage of health care workers. Results of the review suggest that in some contexts where health systems are weak and the coverage of health care poor, elucidating the ways in which factors interact to enhance or challenge sustainability will require understanding from a complex systems approach. Complex systems such as health care settings or communities have several defining characteristics including the tendency to be self-organizing, sensitive to initial conditions, and make non-linear phase transitions (i.e., jump too quickly from one position to another very different position), with interaction effects and feedback [60]. These characteristics will influence whether the sustainability of a potential effective intervention is a simple or complicated process. The complex systems approach enables researchers to be aware of the interactions that occur between components of the intervention itself as well as with the socio-cultural context in which it is implemented and the influence of broader organizational and policy factors present in the setting where the intervention is implemented. In the reviewed literature, only two studies did that. In Zambia, systems thinking was used to evaluate ways to enhance quality improvement in health care delivery [31]. In Rwanda, sustainability was framed as a dynamic question and causal loops used to unearth not only unknown factors but also how other factors combine to negatively shift the evolution of community-based interventions in limited resource settings [61].

### Toward the sustainability of interventions implemented in sub-Saharan Africa: A conceptual framework

For sub-Saharan Africa, while we now have some description of the positive and negative factors that influence the sustainability of interventions, what is not well understood or measured is the relative influence of these factors in particular contexts. Furthermore, little attention has been paid to the dynamic nature of sustainability within and among countries and changes over time. Thus, we recommend the need to develop multi-faceted approaches tailored to context and the broader dynamic and complex health transition ongoing in the region.

Thus, frameworks such as the Dynamic Sustainability Framework (DSF) [16] with its focus on the intervention, the context in which the intervention is delivered, and the ecological systems within which the interventions exist and operate within as well as the consideration of these elements over time is one example that provides unparalleled opportunity to promote sustainability of interventions in sub-Saharan Africa. The fact that DSF incorporates “noise” within health care contexts, advocates for continuous assessment of the local context, while supporting the evolution of an intervention within a changing context also offers the opportunity to assess sustainability as a dynamic process in the region [16]. DSF can also be aligned with alternative existing frameworks currently used in the region like the PEN-3 cultural model with its focus on community dialogue or what Airhihenbuwa [62, 63] refers to as a “polylogue,” whereby attention is paid to language used, expressions (verbal and non-verbal) for communication, and meanings ascribed to what is being discussed. For example, in previous research conducted in the region, we know that words may remain the same but their meanings change as they cross cultural boundaries [64]. We believe that the importance will not always be the *etic* (scientific) accuracy of usage, but rather the *emic* (cultural and community understanding) of what is being discussed [64].

**Fig. 3:**
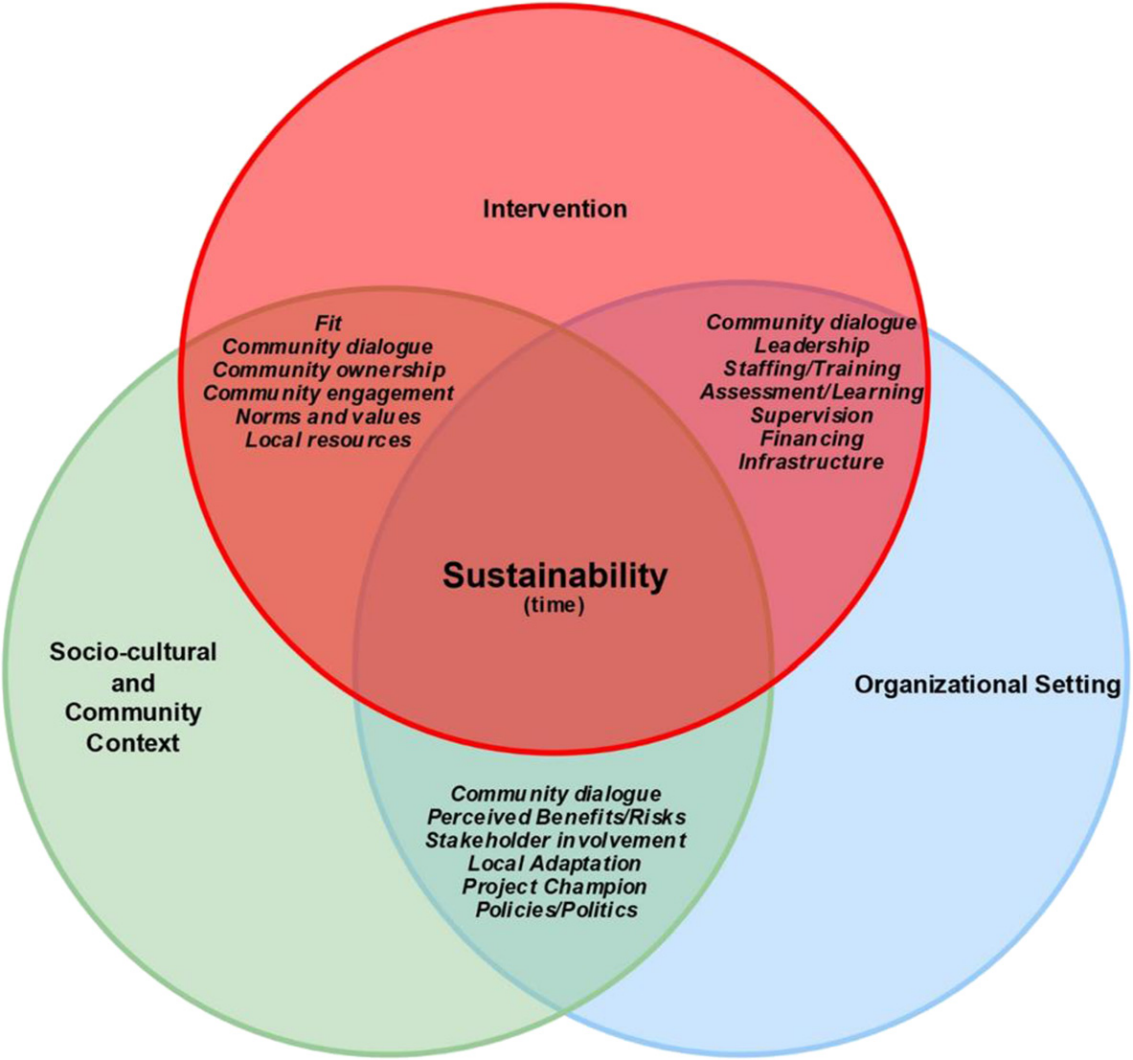
Conceptual framework of sustainability of interventions implemented in SSA

To that end, we propose a comprehensive conceptual framework that broadly maps the terrain of findings from interventions implemented in SSA focused on sustainability while blending aspects of frameworks such as DSF with already existing frameworks on health in the region such as the PEN-3 cultural model. The framework presented in Fig. 3 emphasizes the intersection between the intervention itself and broader socio-cultural and community context within which the intervention is implemented as well as the role of organizational factors in influencing the sustainability of the intervention over time. In doing so, it brings attention to sustainability as a core component embedded within the overall life cycle of an intervention that evolves through time.

The framework also illustrates a number of important lessons for fostering long-term sustainability of interventions implemented in SSA, and they include the following: maintaining a strong fit between core intervention elements and existing socio/cultural and community resources and creating supporting structures between the intervention and the organizational settings while continually fostering community dialogue about the intervention on a daily basis to enhance not only community’s capacity to readily integrate interventions into existing practices but also to address underlying elements at the intervention, socio-cultural/community context, and/or organizational settings that can support or threaten the sustainability of the intervention. The process of engaging in community dialogues also acknowledges that members of communities have a voice that should not be omitted or taken for granted as they play a crucial role with working to sustaining essential elements of implementation that have a strong fit with their context [65]. Deep engagement with communities in dialogue can set appropriate directions for intervention, highlight perceptions about benefits versus risks, and reveal emerging opportunities and constraints arising within the intervention, community, and the broader organizational context that can facilitate sustainability.

Like DSF [16], the framework also demonstrates that sustainability is not a static process. Nestled in the middle with sustainability is time, an unresolved issue that can threaten the potential to sustain any intervention. We find it useful to conceive both sustainability and time as mutually enabling or constraining. Most interventions implemented in SSA usually have a ceiling of 3–5 years because of funding mechanisms and policy logic of research projects, with sustainability of intervention effects assessed 12 months after initial implementation [20]. Yet, in a setting like parts of SSA where resources are limited, where the burden of diseases are growing with no set timeframe, and where infrastructure, including leadership support for implementations, are weak [4, 5, 9, 11, 13], a key point is that sustainability is likely to be affected not only by the intervention characteristics, socio-cultural context, or organizational setting but also by time. The iterative link between these influences thus offers valuable insights on the dynamic process involved with planning for and considering structures embedded within the various levels that will support the continuation of interventions over time [16]. This includes allowing enough time to promote a sense of community ownership, fostering community engagement, paying attention to local norms and values, identifying local resources and local champions of interventions, addressing ethics by ongoing stakeholder discussions, including perceptions of benefits versus risks involved, continuous adaptation to fit local context, staff training and orientation, supervision, ongoing assessment, feedback, and learning, and building sound infrastructure and financing mechanisms to ensure that the promise of sustainability is reached long after initial intervention implementation.

From the perspective of researchers and policymakers, understanding the nature of these intersections will help inform and refine strategies to ensure that interventions are continued for many years thereby facilitating the efficient and effective use of available resources. Use of the proposed conceptual framework will also help to promote and ensure the consideration of key factors that are central to the sustainability of interventions implemented in the region. Although the framework is only hypotheses-generating and not predictive, it can also lay the foundation for the operationalization and measurement of measures and outcomes so as to test which factors are or are not predictors of longer-term sustainability [14].

### Limitations of the study

Although we made every effort to provide a comprehensive review and synthesis of all available literature through the use of a systematic search strategy with critical appraisal of our processes, this review has limitations. First, we only report studies published in English. Second, there are limited data on peer-reviewed evidence-based interventions reporting sustainability outcomes in sub-Saharan Africa. Finally, few studies assessed sustainability over time, which itself was inconsistently defined and applied such that comparisons of outcomes across different settings both within and across countries in sub-Saharan Africa were nearly impossible to examine.

## Conclusion

This is one of the first reviews to comprehensively examine sustainability of health interventions implemented in a region traditionally underrepresented in dissemination and implementation research and for which sustainment of any intervention or program may not be a simple process. Despite the limitations, our findings highlight the need for more literature on the sustainability of evidence-based interventions conducted in sub-Saharan Africa paying close attention to the serious gaps in understanding [22]. We present a conceptual framework to describe the dynamics involved in sustaining interventions implemented in SSA. The framework highlights the linkages between the intervention, the socio-cultural and community context in which it occurs, and the organizational factors in which it operates [16]. Understanding how these linkages intersect over time is important for advancing the research agenda on the sustainability of health interventions in the region. Given the findings of this review, the limitations of available resources and the worrisome data on the growing dual burden of diseases in SSA, it is suggested that at a minimum, plans for sustainability should be incorporated routinely with interventions implemented in the region.
