# Erratum to: ‘Toward the sustainability of health interventions implemented in sub-Saharan Africa: a systematic review and conceptual framework’

**DOI:** 10.1186/s13012-016-0415-5

**Published:** 2016-04-18

**Authors:** Juliet Iwelunmor, Sarah Blackstone, Dorice Veira, Ucheoma Nwaozuru, Collins Airhihenbuwa, Davison Munodawafa, Ezekiel Kalipeni, Antar Jutal, Donna Shelley, Gbenga Ogedegbe

**Affiliations:** 1 Department of Kinesiology and Community Health, College of Applied Health Sciences, University of Illinois Urbana-Champaign, Urbana-Champaign, USA; 2 School of Medicine, New York University, New York, USA; 3 Saint Louis University, Saint Louis, USA; 4 World Health Organization Regional Office for Africa, Brazzaville, Congo; 5 Department of Geography, University of Illinois Urbana-Champaign, Urbana-Champaign, USA; 6 West Virginia University, Morgantown, USA

Unfortunately, the original version of this article [1] contained an error. One of the author’s names was spelt incorrectly. Gbenga Ogedegbe is the correct spelling and is included here. The article will also be updated.
